# Cecal growth factors promote enteric neurosphere formation and hindgut colonization in the avian model

**DOI:** 10.3389/fcell.2025.1681844

**Published:** 2025-12-18

**Authors:** Ádám Soós, Emőke Szőcs, Viktória Halasy, Zsanna Gecse, Fruzsina Mógor, Csenge Jurenka, Katalin Kocsis, Jitka Mucksová, Jiří Hejnar, Nándor Nagy

**Affiliations:** 1 Department of Anatomy, Histology and Embryology, Faculty of Medicine, Semmelweis University, Budapest, Hungary; 2 BIOPHARM, Research Institute of Biopharmacy and Veterinary Drugs, Jílové u Prahy, Czechia; 3 Department of Viral and Cellular Genetics, Institute of Molecular Genetics, Czech Academy of Sciences, Prague, Czechia

**Keywords:** enteric nervous system, neurosphere, neural crest, hirschsprung disease, GDNF, WNT11, endothelin-3, noggin

## Abstract

**Introduction:**

The enteric nervous system (ENS) originates from neural crest cells (NCC) that migrate along the developing gut and differentiate into enteric neurons and glial cells. Disruption of ENS development leads to neurointestinal disorders, such as Hirschsprung disease (HSCR), characterized by aganglionic segments in the distal colon. ENS-derived stem cells (ENSCs), capable of forming multipotent neurospheres, have shown great promise for cell-based therapies. However, optimizing the cell culture conditions and understanding the molecular signals that regulate ENSC development remain unclear. Given the conserved developmental interactions between NCCs and the gut mesenchymal environment in mammals and birds, the avian embryo provides a valuable model for investigating ENS development.

**Methods:**

In this study, we developed and characterized an avian model system for generating enteric neurospheres from transgenic *mCherry*-labeled chick gut tissue.

**Results:**

Addition of GDNF, WNT11, endothelin-3, and the BMP inhibitor Noggin (GWEN medium) resulted in significantly larger and more numerous neurospheres compared to control cultures. Immunostaining showed that GWEN-treated neurospheres contained abundant SOX10+ glial precursors, HU + neurons, and SOX10+/PHOX2B+/HU- progenitors, indicating both differentiation and maintenance of stem cells. When plated on a fibronectin-coated surface in the presence of GDNF, cells from GWEN-treated neurospheres migrated a longer distance and extended more βIII-tubulin + neurites than controls, demonstrating enhanced neurogenic potential. Using *ex vivo* recombination assays and chorioallantoic membrane transplantation, we demonstrate that E12 *mCherr*y+ neurospheres pre-cultured in GWEN medium migrate extensively and form enteric ganglia within host hindgut tissue.

**Conclusion:**

These findings support the neurosphere-forming potential of avian ENSCs and identify ceca-derived signals (GDNF, WNT11, ET-3) and Noggin as potent regulators of ENS progenitor maintenance and differentiation.

## Introduction

The enteric nervous system (ENS) contains a highly complex network of neurons and glial cells embedded in the mesoderm-derived loose connective tissue and smooth muscle layers of the gastrointestinal tract. As a critical regulator of gut function, the ENS orchestrates intestinal motility, glandular secretion, absorption, and immune responses (Furness, 2012). During embryogenesis, the ENS originates from multipotent neural crest cells (NCCs) that develop from the dorsal neural tube and migrate extensively to populate the entire length of the intestine (Nagy and Goldstein, 2017). Disruptions in the migration, proliferation, or survival of these progenitors can result in congenital gastrointestinal disorders, including gastroparesis, esophageal achalasia, and Hirschsprung disease (HSCR) (Burns and Thapar, 2014; Montalva et al., 2023). HSCR is one of the most extensively studied congenital neurointestinal disorders, affecting approximately 1 in 5,000 live births, mostly involving the distal segments of the colorectum, where abnormal NCC development leads to aganglionic regions of variable length. The aganglionic segment lacks motility, resulting in functional obstruction and chronic constipation (Montalva et al., 2023). While surgery is life-saving, over 50% of children have persistent problems, including severe constipation, fecal incontinence, and enterocolitis (Catto-Smith et al., 2007). Cell-based therapy offers the potential to introduce new neurons into the aganglionic region as a novel therapy for this disease (Wilkinson et al., 2015; Montalva et al., 2023).

Multiple studies have reported that human, mouse, rabbit, and swine enteric neural stem cells (ENSCs; the stem/progenitor cells prospectively isolated from the embryonic or postnatal ENS), which arise from the dissociated neuronal plexuses of the intestine, can be cultured, expanded, and transplanted into animal models of enteric neurocristopathies (Lindley et al., 2008; Metzger et al., 2009; Raghavan et al., 2013; Hotta et al., 2016; Hotta et al., 2023; Cheng et al., 2017). In the right culture conditions, ENSCs form spherical aggregates, called enteric neurospheres, which can be maintained for weeks, exhibit multipotency, and differentiate into functional neurons and glia (Cheng et al., 2017). Transplantation of enteric neurospheres into the adult mouse colon has been shown to improve gastrointestinal motility, highlighting the functional integration of these cells within the host and leading to functional recovery and even enhanced survival in HSCR mouse models (Almond et al., 2007; Rollo et al., 2016; Hotta et al., 2016; Majd et al., 2025).

The ENS-forming potential of enteric neurospheres differs significantly depending on their origin. Enteric neurospheres established from embryonic gut have the highest ENS forming potency, containing progenitors capable of robust migration, proliferation, and differentiation into both neurons and glia (Almond et al., 2007; Hotta et al., 2013; McKeown et al., 2017; Nagy et al., 2021; Mueller et al., 2024). In contrast, iPSC-derived neurospheres show moderate to high potency depending on the cell culture protocol and can generate ENS lineages able to integrate into host gut tissues (Barber et al., 2019; Majd et al., 2025). Neurospheres generated from the postnatal ENS have the lowest enteric neuron-forming capacity (Navoly and McCann, 2021; Pan et al., 2024; Rahman et al., 2024), as they consist of lineage-restricted glia and neuron progenitors with limited proliferation and neuronal differentiation, while their accessibility and autologous use make them a promising therapeutic option.

Extensive research over the past few decades has provided important insights into the normal development of the ENS and the causes of HSCR (Goldstein et al., 2013; Montalva et al., 2023). Several morphogens and mesenchymal-derived growth factors, such as glial cell-line derived factor (GDNF) and endothelin-3 (ET-3) have been implicated in ENS development and HSCR pathogenesis (Amiel et al., 2008; Heanue and Pachnis, 2007; Gazquez et al., 2016; Watanabe et al., 2017). Based on mouse and chicken embryo studies, we and others have demonstrated that exposure to the cecum (located at the junction of small and large intestine) mesenchyme is essential for the colonization of the hindgut by enteric neural crest-derived cells (ENCDCs; NCCs that have entered the gut and are committed to the ENS lineage but include undifferentiated progenitors and early differentiating cells). Interestingly, developmentally important morphogens (GDNF, ET-3, or the non-canonical WNTs, particularly WNT5A/WNT11) are expressed at higher levels in the cecum than in the adjacent hindgut segment (Druckenbrod and Epstein, 2005; Nagy and Goldstein, 2006; Nagy et al., 2021; Kovács et al., 2023). Thus, there is a rationale for using ceca-specific growth factors to enrich enteric neural progenitor cells and promote neurosphere formation.

In this study, we describe a cell culture technique for generating enteric neurospheres from ENSCs isolated from transgenic *mCherry* chick embryonic gut tissue. We show that culturing avian enteric neurospheres in the presence of GDNF/WNT11/ET-3 and the BMP4 inhibitor, Noggin (GWEN culture media), significantly increases the number and size of neurospheres. We demonstrate that avian neurospheres are similar to previously reported mammalian neurospheres and contain undifferentiated progenitors, glia, and neurons. Furthermore, when transplanted into the preganglionic chick hindgut and cultured on chorioallantoic membrane, only the GWEN-treated neurospheres differentiate toward enteric neurons, migrate extensively, and form enteric ganglia across the host hindgut.

## Materials and methods

### Experimental animals

White Leghorn-type specific-pathogen-free (SPF) chickens (*Gallus domesticus*) were obtained from a commercial breeder (Biovo Ltd., Mohacs, Hungary). Transgenic *mCherry* chicken embryos were obtained from the Institute of Molecular Genetics of the Czech Academy of Sciences (Trefil et al., 2017). The eggs were incubated at 37.5 °C in a humidified hatching incubator (Heka Brutgerate, TS-7000C), and the age of the embryos was determined by the number of embryonic days (E). All the animal experiments were conducted in accordance with the guidelines of the Institutional Animal Care and Use Committee of Semmelweis University (nr.: 70/2012).

### Histological procedures

For cryosections, tissue samples were fixed in 4% paraformaldehyde (PFA) for 1 h at room temperature, then infiltrated with 15% sucrose overnight at 4 °C, followed by 7.5% gelatine (Sigma, G-2625) in 15% sucrose for 1 h at 37 °C. Gelatine-impregnated tissues were rapidly frozen at −50 °C in 2-methylbutane (Sigma, M32631), and 12 μm cryosections were collected on poly-L-lysine (Sigma, P8920) coated microscope slides.

### Immunofluorescence

Frozen sections were rehydrated in 1x PBS at 37 °C for 10 min and pretreated with 0.1% Triton X-100 (Triton X-100: Sigma, X100-5 ML) in PBS to permeabilize the nuclear and cell membranes. Tissue sections were incubated with primary antibodies (Table 1), diluted in 1% PBS-BSA (bovine serum albumin, Sigma, A9647) for 60 min in a humid chamber at room temperature (RT). Alexa fluor-conjugated secondary antibodies (Goat anti-Mouse IgG, A-11005; Goat anti-Mouse IgM, A-10680; Donkey anti-Goat IgG, A-11055; Donkey anti-Rabbit IgG, A-21207; Goat anti-Mouse IgG1, A-21121; Goat anti-Mouse IgG2b, A-21145; all from Thermo Fisher Scientific) were applied according to the isotype and species specificity of the primary antibodies. Secondary antibodies were diluted 1:200 in PBS and incubated with the sections for 45 min at RT. In the case of double immunofluorescence, the staining method was repeated with the addition of a second primary antibody, followed by an appropriate isotype-specific secondary antibody. The cell nuclei were labeled with DAPI (4,6-diamino-2-phenylindole dihydrochloride; Invitrogen, D1306), and the sections were covered with a water-soluble, non-fluorescent mounting medium (Poly-Aqua Mount, Polyscience Inc., Warrington, PA) and stored at 4 °C.

**TABLE 1:** List of primary antibodies.

Antigen	Cell type	Catalog number	Source	Isotype
SOX10	Neural crest/glia	AF2864	Bio-techne	Polyclonal goat IgG
B-FABP	Glia	Kindly gifted by Thomas Müller	Kurtz et al. (1994)	Polyclonal rabbit IgG
PHOX2B	Neural crest/neuron	AF4940	R&D systems	Polyclonal goat IgG
HU	Neuron	A-21271	ThermoFisher Scientific	Mouse IgG2b
TUJ1	Neuron	SC-80016	Santa Cruz	Mouse IgG2a
HNK1	Neural crest derived cell	MS-1163-P	ThermoFisher Scientific	Mouse IgM
Desmin	Mesenchymal cell	MA5-13259	Acris, Germany	Mouse IgG1
Collagen I	Mesenchymal cell	Kind gift of Dr. Thomas F. Linsenmayer	Swasdison et al. (1992)	Mouse IgG2a
Hsp47	Mesenchymal cell	386,023	Sigma	Mouse IgG2a
Calponin	Smooth muscle cell	CP-93	Sigma	Mouse IgG1

### Generation of enteric neurospheres from chicken hindgut

Low-adherence tissue culture plates were prepared by coating 6-well cell culture dishes (Thermo Scientific, 140,675) with 2 mL of Anti-Adherence Rinsing Solution (StemCell Technologies, 07,010), followed by incubation for 1 h at RT with gentle rocking at 80 rpm. The colorectum of E12 and D0 chicken embryos was dissected under sterile conditions. After washing with PBS supplemented with 100 μg/mL Penicillin-Streptomycin (Pen/Strep; Gibco, 15140122), the solution was aspirated and replaced with 3 mL of Digestion Enzyme Mix (DE), consisting of 2 mL of dispase II (5 U/mL; StemCell Technologies, 07,913) and 2.5 mg of collagenase IX (800 U/mg; Sigma, C9722-50 MG) dissolved in 4 mL of NeuroCult™ Basal Medium (NBM; StemCell Technologies, 05,700). Tissues were enzymatically digested at 37.5 °C for 45 min, with gentle vortexing performed three times during the incubation. Following digestion, the cell suspension was filtered through a cell strainer (mesh size 40 μm; Greiner, 542,040) and gently washed with NBM to a final volume of 20 mL to stop the enzymatic activity.

For the culture medium, 3 mL of NBM was supplemented with NeuroCult™ Proliferation Supplement (Mouse and Rat, StemCell Technologies, 05,701), epidermal growth factor (EGF; StemCell Technologies, 78,006.1) at 40 ng/mL, basic fibroblast growth factor (bFGF; StemCell Technologies, 78,003.1) at 20 ng/mL, and heparin (StemCell Technologies, 07,980) at 4.44 μg/mL (0.0004%). To assess the effect of ceca-specific mesenchymal factors on enteric neural stem cells, the culture medium was further supplemented with 10 ng/mL glial cell line-derived neurotrophic factor (GDNF; R&D Systems, 212-GD-010), 200 ng/mL WNT11 (Origene, TP761904), 250 ng/mL endothelin-3 (ET-3; R&D Systems, 1,162/100U), and 100 ng/mL Noggin (R&D Systems, 6997-NG-025).

Isolated cells were centrifuged at 500 x g for 8 min. The supernatant was carefully aspirated, and the cell pellet was resuspended in 1 mL of growth factor-supplemented culture medium. Cells were seeded into the prepared 6-well low-adherence culture plates at a density of 6 × 10^5^ cells/mL and cultured to form enteric neurospheres for 4–7 days. A 1:1 media change was performed every second day. To assess cell proliferation, 10 µM 5-ethynyl-20-deoxyuridine (EdU; BaseClick) was added to the culture medium 5 h prior to 4% paraformaldehyde (PFA) fixation. EdU incorporation was detected using the Click-Tech EdU Imaging Kit (BaseClick, BCK-EdU488IM100).

### Enteric neurosphere transplantation to aganglionic embryonic chick hindgut

As we described previously (Nagy et al., 2020), neurospheres were transplanted into the isolated preganglionic ceca + hindgut of E5 chick embryos. One neurosphere was transplanted into each of the two ceca buds of the hindgut. To allow the neurospheres to integrate into the aganglionic hindgut mesenchyme before chorioallantoic membrane (CAM) grafting the neurosphere-hindgut recombinants were cultured on the surface of collagen gel matrix prepared by adding 1 N NaOH and 1 mg/mL type I rat tail collagen (BD Biosciences, 354,236) to Dulbecco’s Modified Eagle Medium/Nutrient Mixture F-12 (DMEM/F-12, GlutaMAX™ supplement, ThermoFisher Scientific, 10565018) supplemented with 1% Penicillin-Streptomycin. After 12 h, the explants were removed from the collagen gel and cultured on the CAM of an E9 host chick (*n* = 12 per group) for 7 days as previously described (Nagy and Goldstein, 2006), then processed for immunofluorescence.

### *In vitro* migration assay of neural progenitors from neurospheres

To test the migration capacity of cells from neurospheres, cell aggregates were seeded on the surface of Petri dishes coated with 20 μg/mL fibronectin (Thermo Fisher Scientific, 33010018) diluted in DMEM containing 1% Penicillin/Streptomycin. Emigration of neural progenitors was stimulated with 10 ng/mL GDNF, diluted in DMEM, and monitored for 24 h. Cells were fixed with 4% PFA for 20 min, washed with PBS, and processed for immunocytochemistry. Quantification of ENCDC migration and neurite extension from the spheroids was analyzed with TUJ1 and HNK1 specific immunostaining, measuring the longest neurite projections/most distant cells, with five data points recorded from the border of each spheroid (*n* = 15, three spheroids per group).

### Tissue recombination and organ culture

For chick-*mCherry* chick recombination, E5 (Hamburger-Hamilton stage, HH 25) chick midgut/hindgut was dissected, extending from the ceca to the distal cloaca. The nerve of Remak and the cloaca were removed. This chick’s proximal hindgut was recombined with mid-colon isolated from E12 and D0 *mCherry* chicken embryos. The proximal-distal orientation of the hindgut and colon was maintained in the recombination. To allow the gut tissues to adhere, recombinants were explanted onto a collagen gel matrix surface (as described above). After 12 h, a second collagen gel layer was applied to fully embed the tissues within the 3D matrix and culture them for 72 h.

### Evaluation of samples

Samples were analyzed by fluorescence microscopy (Nikon Eclipse E800, Zeiss Axiovert 135), and confocal laser scanning microscopy (Zeiss LSM 780, LSM 900, Olympus FV3000, FV4000). The micrographs were processed using the ImageJ software and Adobe Photoshop CC 2020.

The DAPI/Hu double-stained confocal images were analyzed using a custom Fiji (ImageJ 1.54c) macro with Java 1.8.0_322. We identified neurospheres as ROIs by segmenting the Hu channel image. A preprocessing of inversion, thresholding, morphological filtering (20–30 px radius), and watershedding, was followed by automatic particle counting (1,000–50,000 px^2^, circularity 0.5–1.0), and a manual review. Within each neurosphere, we isolated cell nuclei based on the DAPI channel image. An alternative preprocessing of background subtraction (rolling = 30 px), thresholding (0–13 a. u.), median-filtering (radius = 1 px), and watershedding, was again followed by automatic particle counting (10–1,000 px^2^, circularity 0.3–1.0). Finally, we assessed Hu-positivity in cells by measuring color intensity in the Hu channel. Cells with >10 a. u. in their nuclei were counted as Hu+, otherwise as Hu-. Measurements were then integrated into counts per neurosphere. ROI and overlay images were saved for quality control.

A modified version of the DAPI/Hu analysis macro was adapted to process multichannel images, enabling parallel classification of fluorescence positivity in the green, red, and far-red channels for the cells of each neurosphere. ROIs were drawn manually using the PHOX2B/green channel. All images were background-subtracted (rolling = 30 px) and converted to 8-bit. Within each ROI, DAPI + nuclei were isolated by thresholding (0–50 a. u.), binarization, and watershed separation, retaining particles 0.001–1,000 px^2^ (circularity 0.1–1.0). Mean fluorescence intensity for each nucleus was measured in the three additional channels (PHOX2B- green, Sox10 - red, EdU - far-red). Thresholds were 10 a. u. (green, far-red) and 30 a. u. (red) to classify nuclei as positive or negative. Results were exported automatically to CSV. ROI and overlay images were saved for quality control.

Statistical analyses were performed in GraphPad Prism (v10.6.1). Depending on the data distribution and experimental design, group comparisons were performed using either Kruskal–Wallis tests or one-way ANOVA, followed by Dunn’s or Tukey’s *post hoc* tests, respectively. For pairwise comparisons, Mann-Whitney tests were applied. Holm correction was used to adjust p-values for multiple testing. Statistical significance was set at p < 0.05, with the following thresholds applied throughout: **p < 0.01, ***p < 0.001, ****p < 0.0001. Error bars represent the standard error of the mean (SEM).

## Results

### Spatiotemporal distribution and differentiation of SOX10+/PHOX2B + enteric neural crest-derived cells (ENCDC) during chicken hindgut development

**FIGURE 1:**
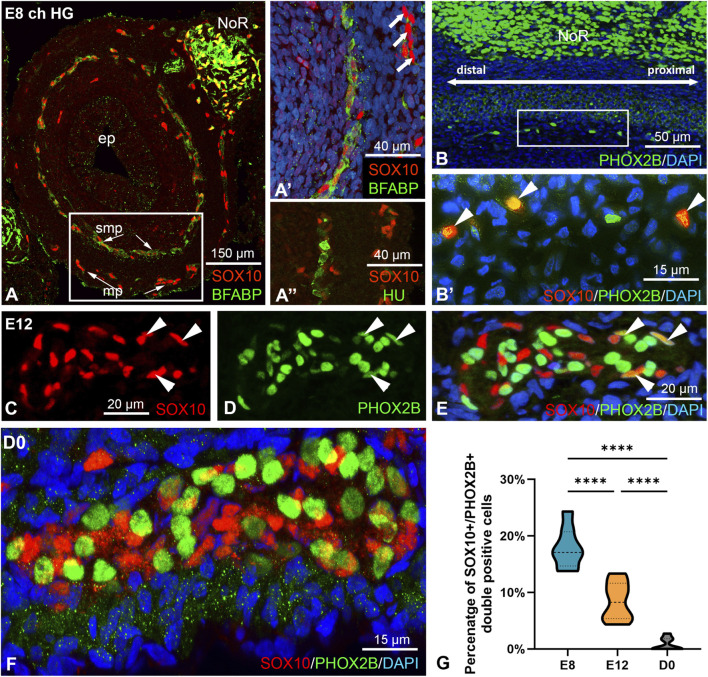
Distribution of SOX10+/PHOX2B+ enteric neural crest-derived cells (ENCDC) during development of the chicken hindgut. Embryonic day (E) 8 hindgut **(A-B′)**. ENCDCs in the cross-section of the mid-hindgut level show SOX10, B-FABP **(A,A′)**, and PHOX2B **(B,B′)** immunoreactivity. Most of the SOX10+ cells (red) in the submucosal plexus are also B-FABP+ (green), representing the enteric glia cell population **(A)**. The outlined area in A is magnified in **(A’)**. At this developmental stage, the majority of the myenteric plexus forming cells are still single SOX10 immunoreactive ENCDCs (arrows). HU + neuron **(A″)** and SOX10+/B-FABP+ glial differentiation is present only in the submucosal layer **(A′)**. At the distal hindgut level, wavefront ENCDCs were localized in a narrow strand corresponding to the presumptive submucosal plexus **(B,B′)** and co-express SOX10 and PHOX2B (**(B′)**, arrowheads). The outlined area in **(B)** is magnified in **(B’)**. E12 hindgut **(C–E)**. High power images of SOX10+/PHOX2B+ **(C–E)** co-expression confirm the presence of ENCDCs (arrowheads) in chicken myenteric ganglia. Magnified view of colon myenteric ganglia at hatching (D0) **(F).** Differentiated ENCDCs in the enteric ganglia express either SOX10 in glia or PHOX2B in the neuronal lineage. Only a small percentage of SOX10+ cells are positive for PHOX2B. Diagram showing the proportion of SOX10+/PHOX2B+ ENCDCs in E8, E12 and D0 in the large intestines **(G)**. Measurements were taken from eight separate colons (n = 8) for each stage. ****P < 0.0001. ep, epithelium; mp, myenteric plexus, NoR, nerve of Remak; smp, submucosal plexus.

To characterize ENCDC differentiation in the developing chicken hindgut, we examined the expression patterns of undifferentiated ENCDC markers SOX10 and PHOX2B, the glia marker brain-specific fatty acid binding protein (B-FABP) and the enteric neuron marker TUJ1 (beta-III tubulin) and HU across different developmental stages using immunofluorescence labeling. As demonstrated previously (Burns and Le Douarin, 2001; Nagy et al., 2012), at embryonic day 8 (E8) in the chicken hindgut, ENCDCs colonize the submucosal plexus and migrate ahead of the myenteric plexus. By this developmental stage, their migration is nearly complete, and form two distinct nervous plexuses - composed of undifferentiated ENCDCs, neurons, and glial cells expressing SOX10, PHOX2B, HU, B-FABP (Figures 1A,A’,A”; Supplementary Figure S1) - throughout the hindgut, extending to the cloaca. SOX10+ ENCDCs were observed within the submucosal and myenteric regions of the mid-hindgut (Figures 1A,B’). In the submucosal plexus, a large proportion of SOX10+ cells co-expressed B-FABP, indicating commitment to the glial lineage (Figures 1A,A’). In the myenteric plexus, however, neuron (HU+; Figure 1A”) and glia (B-FABP+; Figure 1A’) specific markers were largely absent, suggesting that differentiation is initially restricted to the submucosal plexus of the mid-hindgut. Longitudinal sections of the E8 hindgut were also stained with SOX10 and PHOX2B. At the distal hindgut level, the leading wavefront of ENCDCs formed a narrow strand corresponding to the presumptive submucosal plexus. These cells co-expressed SOX10 and PHOX2B (Figures 1B,B’ arrowheads), indicating the existence of bipotent progenitor ENCDCs. By E12, immunofluorescence revealed only a few SOX10/PHOX2B double immunopositive cells that lacked both B-FABP and HU immunoreactivity (Supplementary Figure S1, arrowheads). Differentiating enteric glia and neurons were identified by co-expression of SOX10+/B-FABP+ and PHOX2B+/HU+, respectively (Supplementary Figure S1). At hatching (Day 0; D0), SOX10 expression was restricted to glial cells, while PHOX2B was present in the SOX10- neuronal cell population (Figure 1F). Most cells had completed glia and neuron differentiation by this stage (Supplementary Figure S1). Quantitative analysis across developmental stages (Figure 1G) demonstrated a progressive decline in the proportion of SOX10+/PHOX2B+ cells from E8 to D0. This supports a model in which bipotent ENCDCs progressively differentiate into either glial (SOX10+/B-FABP+) or neuronal (PHOX2B+/HU+) lineages during hindgut ENS development.

### Recombination experiments demonstrate stage-dependent contribution of ENCDCs to hindgut ENS formation

**FIGURE 2:**
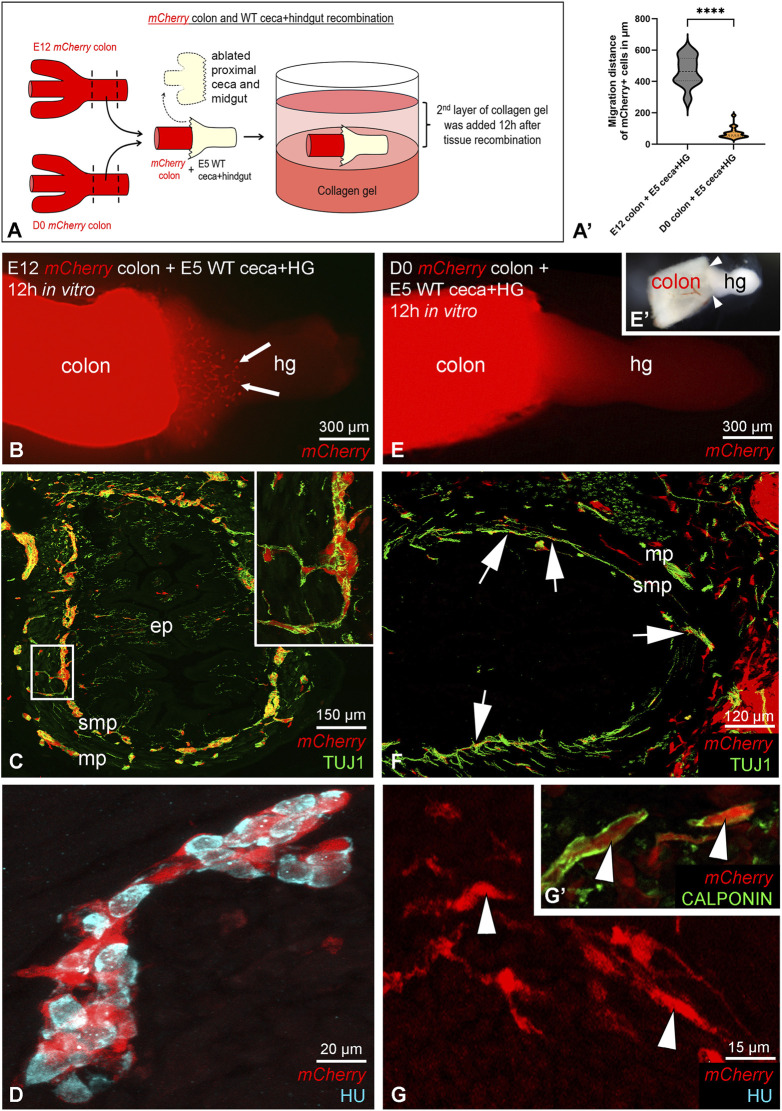
Chick - *mCherry* chick recombination confirm the ENCDCs contribution to the colon ENS. Schematic illustration for intestinal organ culture assays to test the ENS forming ability of ENCDCs from late developmental stages **(A)**. Quantification of mCherry+ cell migration into the cecal and hindgut regions after 12 h of culture, comparing E12 and D0 colons **(A′)**. ****P < 0.0001, n = 21, Mann-Whitney test. Preganglionic E5 chicken hindgut was recombined with E12 **(B)** and D0 **(E)** *mCherry* chicken colon, cultured 72 h in collagen gel, and then examined by immunofluorescence. Brightfield image of the recombinants after 12 h incubation **(E′)**. Arrows show the *mCherry*+ cells originating from E12 colon **(B)**. The red fluorescence reveals E12 *mCherry* chicken colon-derived TUJ1+ neurons in cross-section of the chicken proximal hindgut as they form the submucosal and myenteric plexus of the ENS **(C)**. The outlined *mCherry*-derived TUJ1+ ganglia are magnified in the inset. Magnified view of a single submucosal ganglion: Immunofluorescence staining confirms that HU+ enteric neurons are of E12 *mCherry* colon origin **(D)**. Double immunofluorescence with TUJ1 confirms that some of the *mCherry*+ cells in the chicken hindgut recombined with D0 *mCherry* colon are associated with the nerve fibers (arrows), but do not contribute to enteric ganglia in the hindgut **(F)**. Few *mCherry*+ ramified and spindle-shaped cells are scattered throughout the aganglionic proximal hindgut (**(G)**, arrowheads) and express smooth muscle cell specific calponin (**(G′)**, arrowheads). ep, epithelium; hg, hindgut; mp, myenteric plexus; smp, submucosal plexus.

Several groups have demonstrated that the ability of ENCDCs to colonize the preganglionated gut regions is restricted to a developmental time window, with younger donor ENCDCs exhibiting higher colonization potential (Care et al., 1992; Hotta et al., 2010; Zhang et al., 2019). Our findings using quail-to-chick grafting experiments confirmed that this capacity declines rapidly with donor age. Beginning 2–3 days after the initial ENCDC wavefront cells colonize the hindgut, after which both their number and developmental potential rapidly decline (Zhang et al., 2019). To determine the ENS-forming capacity of ENCDCs from late developmental stages, we performed organotypic culture assays using chick-*mCherry+* tissue recombination (Figures 2A,E’). After ablation of the midgut and proximal end of the ceca the preganglionic E5 chicken distal end of the ceca + hindgut was recombined with either E12 or D0 *mCherry+* chicken colon segments, cultured *ex vivo* for 72 h in a 3D collagen matrix and analyzed by immunofluorescence (Figure 2B). Cross sections of E5 hindgut + E12 *mCherry+* chicken colon recombinants stained with anti-βIII-tubulin (TUJ1) show numerous *mCherry*+ neurons throughout the proximal hindgut. These cells formed distinct ganglionic structures within both the submucosal and myenteric plexuses, indicating successful migration, integration, and differentiation of the donor colon-derived ENCDCs (Figure 2C). High-magnification analysis of individual ganglia further confirmed that HU immunoreactive neurons were derived from *mCherry*+ donor tissue (Figure 2D), supporting the neurogenic potential of E12 ENCDCs in a permissive host environment. In contrast, recombination with D0 *mCherry* colon showed a limited contribution to the E5 hindgut ENS (Figure 2E). Although some *mCherry*+ cells were observed in close association with TUJ1^+^ nerve fibers (Figure 2F), they did not integrate into enteric ganglia, suggesting a reduced capacity for neuronal differentiation at this developmental stage. Moreover, only a few scattered *mCherry*+ cells, identified as calponin immunoreactive smooth muscle cells, were present within the aganglionic mesenchymal layer of the proximal hindgut (Figures 2G,G’). These findings indicate that ENCDCs from E12 colon retain ENS-forming potential, while those from D0 colon exhibit restricted migratory capacity and no ENS-forming capabilities. This data shows a developmental decline in the plasticity and functionality of ENCDCs.

### Characterization and differentiation of chicken enteric neurospheres in response to ceca-specific growth factors

**FIGURE 3:**
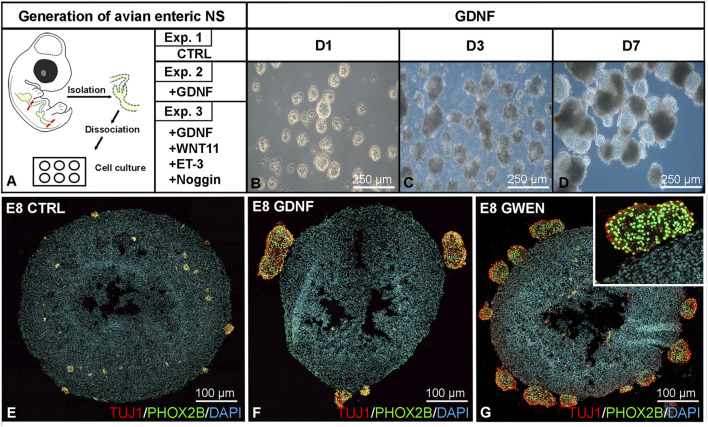
Isolation, characterization and differentiation of chicken enteric neurospheres. Schematic figure of avian enteric neurosphere generation **(A)**. Brightfield images show examples of neurospheres generated from E8 ENSCs that emerged 1, 3, and 7 days in GDNF-containing medium **(B–D)**. Representative immunofluorescence images of 7-day-old neurospheres generated from E8 chicken intestine **(E–G)**. Samples were cultured under three different conditions: Exp.1: control medium (**(E)**, CTRL), Exp. 2: GDNF containing cell culture media (**(F)**, GDNF), and Exp.3: GWEN (**(G)**, ceca-derived mesenchymal growth factors: GDNF, WNT11, ET-3, Noggin) supplemented media. Immunofluorescence of cross-sections confirms the presence of neurons expressing TUJ1, and neural crest cells expressing PHOX2B. Compared to CTRL and GDNF-only conditions, GWEN treatment resulted in a robust increase in TUJ1+/PHOX2B+ cell aggregates on the surface of the neurosphere and single Phox2b+ cells in the center of the aggregates. The inset shows a magnified view of a PHOX2B+/TUJ1+ aggregate, indicating enhanced differentiation of neural crest derivatives **(E–G)**. Scale bar = 100 µm. CTRL, control; NS, neurosphere.

Our current understanding of the developmental mechanisms regulating hindgut ENS formation is primarily based on studies in mouse and chicken embryos, which emphasize the critical role of ceca-derived mesenchymal growth factors such as GDNF, WNT11, ET-3, and BMP4 in hindgut ENS formation (Druckenbrod and Epstein, 2005; Nagy and Goldstein, 2006; Nagy et al., 2021; Kurtz et al., 1994). While generating mouse enteric neurospheres from embryonic and postnatal gastrointestinal tissues is a well-established method, the generation of chicken-derived enteric neurospheres has not yet been reported. We applied mouse-based enteric neurosphere cell culture protocols (Metzger et al., 2009; Hotta et al., 2016; Nagy et al., 2018) to assess the neurogenic and migratory potential of chicken ENS-derived enteric neural stem/progenitor cells (ENSCs) and determine how exposure to embryonic ceca-derived growth factors affects their formation and differentiation. Following dissection and enzymatic dissociation of E8 chicken intestines (Figure 3A), cells were cultured under non-adherent conditions for 7 days to form spheroids, as observed in brightfield images at 1, 3, and 7 days of culture (Figures 3B–D). To assess the differentiation potential, spheroids were cultured under three different conditions: control medium (CTRL), medium supplemented with 10 ng/mL GDNF, and a combination of ceca-derived mesenchymal growth factors including 10 ng/mL GDNF, 200 ng/mL WNT11, 250 ng/mL ET-3, and 100 ng/mL Noggin (GWEN). Immunofluorescence staining was performed after 7-days in culture to evaluate neuronal and neural crest lineage differentiation using neuronal markers TUJ1 and HU, PHOX2B, and SOX10 specific antibodies for ENCDCs (Figures 3E–G; Supplementary Figure S2). While control spheroids displayed limited numbers of TUJ1+/PHOX2B+ cells (Figure 3E), addition of exogenous GDNF recombinant protein induced ENDCDs to form aggregates on the surface of the spheroids (Figures 3B–D,F; Supplementary Figure S2B–B”’), whereas GWEN-treated cultures showed a marked increase in peripheral aggregates (neurospheres) of TUJ1 and PHOX2B double-positive and HU+ cells (Figure 3G; Supplementary Figure S2C–C”’). These neurospheres were located at the spheroid surface. High-magnification imaging confirmed the co-expression of TUJ1 and PHOX2B (Figure 3G inset), suggesting enhanced neurosphere formation and neuronal differentiation of ENCDCs under GWEN conditions (Supplementary Figure S2C). Interestingly, the addition of Noggin or WNT11 to GDNF (Supplementary Figure S2D–D”’,F–F”’) did not alter the immunophenotype compared with GDNF treatment alone, suggesting that BMP inhibition or WNT stimulation did not influence neurogenic differentiation under these conditions. In contrast, combined GDNF and ET-3 treatment (Supplementary Figure S2E–E”’) significantly increased the presence of PHOX2B+/SOX10+ undifferentiated cells within the neurospheres.

Quantitative analysis revealed distinct effects of individual and combined growth factors on ENSC behavior within chicken intestinal neurospheres (Supplementary Figure S3). GWEN and GDNF + ET-3 treatments significantly increased the number of neurospheres formed compared with control and GDNF (Supplementary Figure S3A). The number of HU+ neurons/spheroid was higher in GDNF, GDNF + WNT11, and GDNF + Noggin compared to the control (Supplementary Figure S3B), which shows, that neuron differentiation is increased per spheroid in these cases. Analysis of cell proliferation showed that EdU incorporation significantly increased in GWEN-treated neurospheres (Supplementary Figure S3C), demonstrating that ceca-derived growth factors promote mitogenic activity in ENSCs. Similarly, the proportion of PHOX2B+ cells - a marker of committed neuronal differentiation - was lowest in GWEN and GDNF + ET-3 cultures (Supplementary Figure S3D). In contrast, addition of WNT11 or Noggin to GDNF did not significantly alter progenitor and neuronal proportions relative to GDNF alone (Supplementary Figure S3D–F). Importantly, the GWEN supplement significantly increased the proportion of PHOX2B+/SOX10+ bipotent progenitors (Supplementary Figure S3E) and the number of actively proliferating PHOX2B+/SOX10+/EdU + cells (Supplementary Figure S3F), supporting the synergistic effect of WNT11, ET-3, and Noggin with GDNF in maintaining and expanding undifferentiated ENCDCs as described previously *in vivo* in the chicken embryo (Nagy and Goldstein, 2006; Kurtz et al., 1994). In summary, these results demonstrate that while GDNF alone supports basal neurosphere formation, the addition of the GWEN supplement strongly enhances the proliferation and early neuronal differentiation of SOX10+/PHOX2B+ progenitors *in vitro*. Supplementary Table S1 presents the detailed statistical comparisons corresponding to Supplementary Figure S3, summarizing the results of each treatment.

**FIGURE 4:**
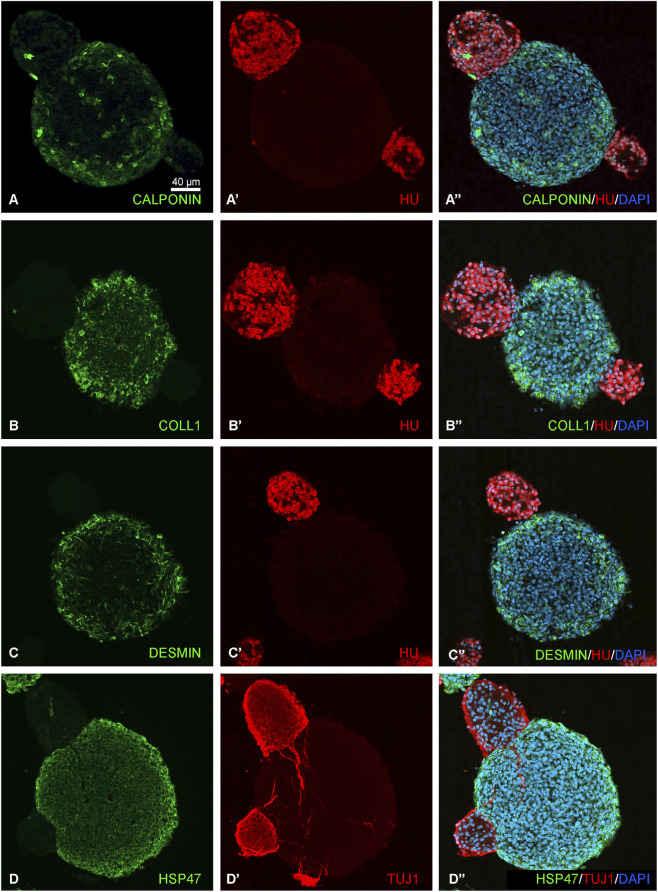
Spheroids contain heterogeneous cell populations expressing smooth muscle, mesenchymal, and neuronal markers. Double immunofluorescence staining of GWEN-treated E12 spheroids showing markers of non-neuronal and neuronal lineages **(A–Dʺ)**. Calponin **(A–Aʺ)** labels smooth muscle cells, while collagen type I **(B–Bʺ)**, desmin **(C–Cʺ)**, and HSP47 **(D–Dʺ)** highlight mesenchymal cells within the spheroid core. Co-staining with the pan-neuronal marker HU **(Aʹ–Cʺ)** or TUJ1 **(Dʹ–Dʺ)** reveals distinct neuronal compartments, referred to as neurospheres. Nuclei are counterstained with DAPI.

To characterize the cellular composition of GWEN-treated spheroids, we performed double immunofluorescence staining for mesenchymal, smooth muscle, and neuronal markers (Figure 4). Calponin-positive cells were distributed primarily at the periphery of the spheroids, indicating smooth muscle differentiation (Figure 4A–A”). Collagen type I (Figure 4B–B”), desmin (Figure 4C–C”), and HSP47 immunoreactivity (Figure 4D–D”) were detected within the spheroid cores, highlighting populations with mesenchymal and fibroblast-like phenotypes. Double-staining with the pan-neuronal markers HU or TUJ1, revealed distinct neuronal clusters adjacent to or partially overlapping with the non-neuronal compartments. These findings demonstrate that E8 hindgut-derived spheroids contain multiple differentiated cell types, including smooth muscle, mesenchymal, and neuronal cells, recapitulating key features of the enteric microenvironment *in vitro*.

### GWEN treatment enhances neuronal differentiation and migration of E12 chicken enteric neurospheres

**FIGURE 5:**
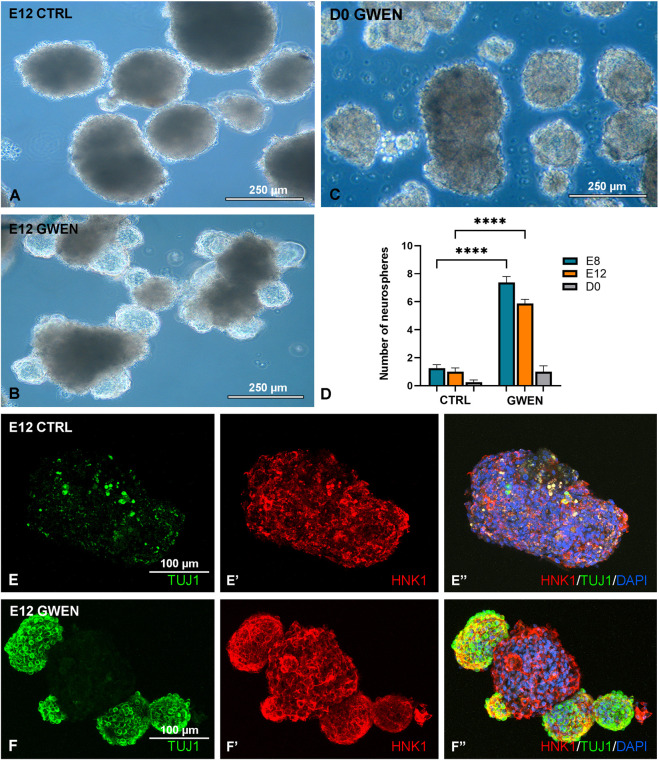
GWEN promotes ENS-derived neurosphere outgrowth and migration *in vitro*. Representative brightfield images of spheroids derived from E12 or D0 colonic tissue cultured in control (CTRL) cell culture media or with GWEN-supplement **(A–C)**. E12 control (CTRL) spheroids show compact morphology **(A)**. E12 GWEN-treated spheroids show the brighter cell aggregates (neurospheres) on their surface **(B)**. D0 GWEN spheroids remain largely compact with no neurospheres **(C)**. Quantification of the neurospheres on spheroids shows a significant increase in E12 GWEN-treated cultures compared to control and other developmental stages (****P < 0.0001, n = 8, One-way ANOVA) **(D)**. Immunofluorescence staining of E12 control (CTRL) and GWEN-treated neurospheres **(E–Fʺ)**. E12 CTRL spheroids exhibit compact organization with limited TUJ1+ immunoreactivity **(E–Eʺ)**. Addition of GWEN-supplement induced ENSCs to form HNK1+/TUJ1+ neurospheres on the surface of the spheroids **(F–F″)**.

Transgenic chicken lines that ubiquitously express green fluorescent protein or mCherry provide powerful tools for developmental biology, enabling precise fate mapping of NCCs, lineage tracing of ENCDCs, and tissue grafting experiments (McGrew et al., 2004; Nagy et al., 2012). To clearly identify and characterize the neurosphere-derived cells in the transplanted colorectum and definitively trace their migration, enteric neurospheres were generated from *mCherry*-labeled ENS tissue. Cells isolated from E12 and D0 wild-type and *mCherry+* chicken small and large intestines were cultured under standard or GWEN-supplemented cell culture media. Similar to E8 intestines, wild-type and *mCherry+* enteric neurospheres formed robustly from both small and large intestines, with tissue pooled from 5–12 embryos per experiment and repeated across eight independent cultures. Under standard conditions and GWEN treatment, wild-type chicken E12-and D0-derived tissue aggregates exhibited compact, spherical morphologies (Figures 5A–C). In contrast, E12 spheroids cultured in the presence of GWEN supplement formed large neurospheres on the surface of the central spheres, indicating the specific developmental response to GWEN treatment. Notably, spheroids generated from D0 intestines did not form neurospheres, even with GWEN supplementation (Figure 5B), suggesting a developmental decline in neurogenic potential. Quantitative analysis confirmed the significant increase in neurosphere formation in GWEN-treated cultures compared to controls (Figure 5D; p < 0.001, n = 12). Immunocytochemical characterization of control spheroids showed they contain few NCC-specific HNK1 immunoreactive cells that co-express the neuron-specific β-III-tubulin (Figure 5E–E”). In contrast, robust neuronal differentiation, as indicated by TUJ1 immunoreactivity, was only evident in GWEN-treated spheroids, where TUJ1+ neurons localized specifically to the neurospheres (Figures 5F,F”).

**FIGURE 6:**
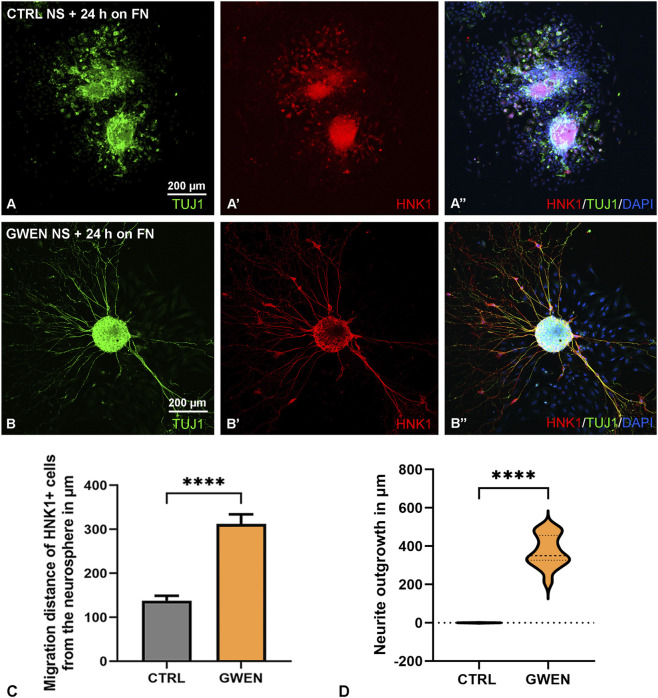
GWEN enhances neuronal differentiation and neurite extension from ENS-derived neurospheres. E12 control and GWEN neurospheres cultured on fibronectin (FN) coated surface for 24 h **(A–Bʺ)**. CTRL neurospheres show no neurite outgrowth and emigrated HNK1 immunoreactive cells **(A–Aʺ)**. GWEN-treated neurospheres exhibit robust radial neurite extension with overlapping TUJ1+ and HNK1+ projections extending from the core **(B–Bʺ)**. Nuclei are counterstained with DAPI. Quantification of HNK1+ cell migration distance from the neurosphere demonstrates significantly enhanced migration in GWEN-treated cultures compared to control **(C)** (****P < 0.0001, n = 15, Mann-Whitney test). Violin plot showing increased neurite outgrowth (in µm) in GWEN-treated E12 neurospheres compared to controls (****P < 0.0001, n = 15, Mann-Whitney test) **(D)**.

To determine the migratory and differentiation potential, E12-derived chicken neurospheres were plated on fibronectin-coated surfaces in the presence of GDNF (Figure 6). After 24 h HNK1+/TUJ1+ cells radially dispersed from control spheroids (Figure 6A–A”), while GWEN-treated neurospheres showed extensive neurite-like projections labeled with TUJ1, indicating active neuronal migration and fiber outgrowth (Figure 6B–B”). Quantification confirmed a significant increase in migration distance of HNK1+ ENCDCs (Figure 6C) and TUJ1+ fiber projections in GWEN-treated cultures (Figure 6D), demonstrating that GWEN growth factors strongly promote both migration and neuronal differentiation in chicken ENSCs. This is the first experimental evidence that chicken enteric neurospheres can be generated *in vitro* and that their growth, differentiation, and migratory potential are enhanced by specific ceca-derived growth factors.

### mCherry+ chicken enteric neurospheres cultured in the presence of GWEN supplement exhibit robust cell migration and ENS formation following transplantation to the preganglionic hindgut

**FIGURE 7:**
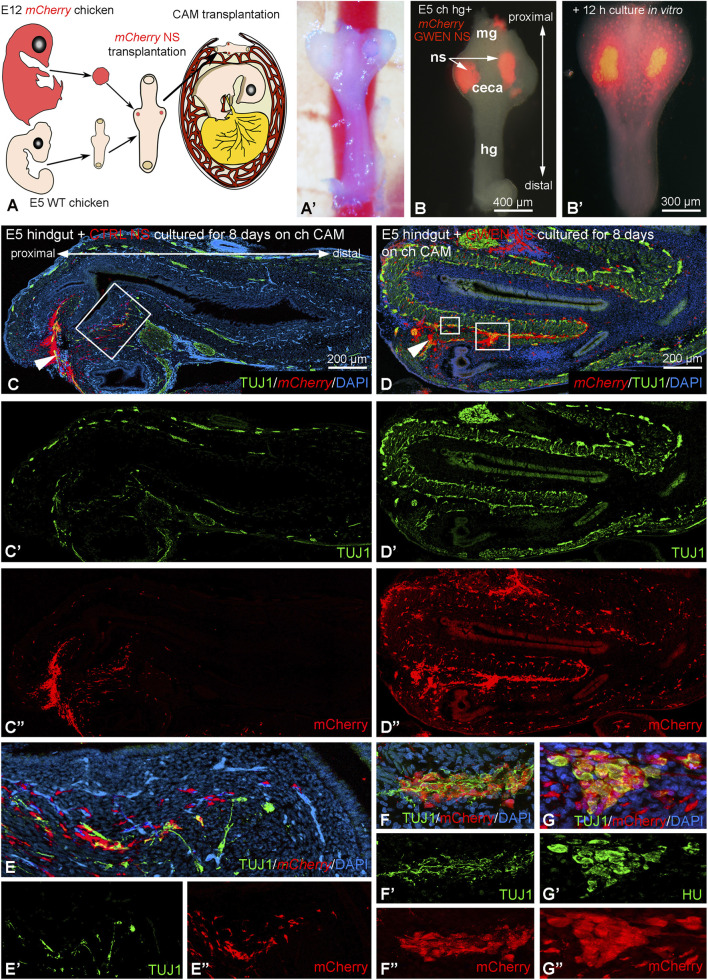
Chicken enteric neurospheres cultured in the presence of GWEN media supplement exhibit increased cellular migration and ENS formation following transplantation into the preganglionic hindgut. Enteric neurospheres from E12 *mCherry* chicken intestine were implanted into E5 wild-type chicken ceca and then grafted onto the chorioallantoic membrane (CAM) of host E8 chick embryos **(A).** Wholemount image of the CAM graft **(A′)**. One or two *mCherry* neurospheres were implanted into each ceca bud and cultured in a 3D collagen to ensure the adherence of the recombinant tissues **(B)**. After 12 h in culture, many *mCherry* expressing cells had emigrated from the neurospheres and colonized the ceca mesenchyme **(B′)**. Neurospheres maintained in the presence of control media and cultured on the CAM for 8 days exhibit limited cell migration in CAM grafts. Arrowhead shows the site of neurosphere transplantation in longitudinal sections **(C)**. Individual immunofluorescence images showing TUJ1+ nerve fibers **(C′,D′,E′)** and mCherry expression **(C″,D″,E″)**. Note that **(E)** represents a magnified view from the boxed area in **(C)**. A similar experiment using embryonic neurospheres cultured in GWEN-media supplement **(D–D″)** reveals robust *mCherry* + ENCDC migration throughout the hindgut to form submucosal and myenteric plexuses that express neuron-specific TUJ1 **(F–F′)** and HU antigens **(G–G″)**. hg: hindgut; mg: midgut; ns: neurosphere.

To examine the ENS forming potential of chicken enteric neurospheres and the effect of GWEN supplementation on their migration and hindgut colonization, we performed chorioallantoic membrane (CAM) transplantation experiments using embryonic *mCherry+* gut-derived neurospheres. Enteric neurospheres derived from E12 *mCherry+* transgenic chicken intestines were implanted into the cecal region of E5 wild-type chicken ceca + hindgut explants and grafted onto the CAM of E9 host embryos (Figures 7A,A’). To ensure tissue integration, recombinants were cultured in a 3D collagen gel matrix (Figure 7B). Within 12 h, numerous *mCherry*+ cells were observed emigrating from the neurospheres into the surrounding cecal mesenchyme, indicating active cell migration (Figure 7B’). Control neurospheres maintained in standard neurosphere culture media exhibited limited cell dispersion following transplantation (data not shown). After 8 days of incubation on the CAM, control neurosphere-derived *mCherry*+ cells remained largely restricted to the site of implantation with minimal evidence of migration into the host tissue (Figure 7C–C”,E–E”). In contrast, neurospheres cultured in GWEN-supplemented medium prior to transplantation demonstrated robust migration throughout the hindgut CAM explant, forming ganglia within both the submucosal and myenteric plexuses (Figure 7D–D”). Immunofluorescence staining confirmed that these cells expressed the pan-neuronal markers TUJ1 (Figure 7F–F”) and HU (Figure 7G–G”).

## Discussion

Although the histogenesis and development of the ENS have been studied in a number of animal model systems, the chicken embryo has proven to be one of the most suitable for ENS-related developmental biology studies (Goldstein and Nagy, 2008). Avian microsurgery experiments demonstrate that ENCDCs, particularly those originating from the vagal neural tube level, exhibit superior migratory and colonization capacities, making them optimal progenitor sources for ENS repair (Burns and Le Douarin, 2001; Nagy et al., 2012). The migration, proliferation, and differentiation of ENCDCs rely on reciprocal interactions between NCCs and the gut mesenchymal microenvironment. Transcriptomic and avian lineage tracing studies also confirmed that exposure to the ceca mesenchyme is critical for the colonization of the hindgut by ENCDCs, as the expression of developmentally important molecules for the ENS, such as WNT5a, WNT11, BMP4, GDNF, and ET-3, are significantly higher in the cecum compared to other regions of the gut (Leibl et al., 1999; Young et al., 2001; Nagy and Goldstein, 2006; Nagy et al., 2021).

Optimization of cell culture conditions is critical for the successful generation of enteric neurospheres. Growth factors that orchestrate embryonic ENS development-promoting the proliferation, guiding the migration, and directing the differentiation of ENCDCs-often exhibit distinct or even opposing effects when applied to postnatal ENS-derived progenitors. Early ENSC studies frequently adopted media formulations from CNS neurosphere protocols, including supplementation with EGF and bFGF, largely by analogy rather than based on ENS-specific optimization (Chen et al., 2023). Several reports indicate that fetal and postnatal ENSCs can be maintained and expanded in self-renewal media that do not contain EGF, and that these conditions support glial multipotency and neurogenic potential in gut-derived NCCs (Joseph et al., 2011; Bixby et al., 2002). In other experimental settings, EGF and bFGF have been added to promote the proliferation of postnatal mammalian enteric neuronal progenitors (Metzger et al., 2009). These findings suggest that the effects of EGF and bFGF on ENSCs are highly context-dependent, and that media recommended for proliferation may not be optimal for neuronal differentiation.

Growth factors known to orchestrate embryonic ENS development, promoting the proliferation, guiding migration, and differentiation of ENCDCs often exhibit distinct or even opposing effects when applied to postnatal ENS-derived progenitors, highlighting an unresolved and puzzling effect in growth factor responsiveness after birth. Exposure to GDNF significantly increases the size and cell number in mouse embryonic enteric neurospheres (McKeown et al., 2017), but shows little or no effect on the density or proliferation of postnatal ENSC cultures (Cheng et al., 2016). WNT signaling inhibits neuronal differentiation during embryonic ENS development (Nagy et al., 2021), while it stimulates proliferation and enhances neurogenesis in mammalian postnatal ENSC cultures (Zhang et al., 2017). Furthermore, ET-3 stimulates ENCDC proliferation and prevents neuronal differentiation only in early embryonic stages but has no effect on postnatal ENSC cultures (Nagy and Goldstein, 2006; Cheng et al., 2016; Carreón-Rodríguez et al., 2017). Altogether, these data suggest that the culture requirements of ENSCs remain incompletely defined, and no general consensus has been reached on optimal media for their isolation, self-renewal, or differentiation.

The main objective of our study was to take advantage of the avian embryo model system to culture ENS cells (ENSCs, enteric glia, and neurons) and generate enteric neurospheres from wild-type and transgenic chicken embryos to test how ENSCs integrate into experimental aganglionic colon. Importantly, we demonstrate that exposure to GDNF, WNT11, and ET-3, all of which are highly expressed in the ceca, and the inhibition of BMP4 with Noggin enhances neurosphere formation. This cocktail also promotes the integration and differentiation of ENSCs into glial and neuronal phenotypes better than control conditions. Furthermore, we show that the developmental stage of the donor tissue critically affects the neurogenic potential of ENCDCs, with embryonic (E12) cells preserving greater ENS-forming ability than postnatal (D0) cells.

Our initial step was to analyze by immunofluorescence the presence of undifferentiated SOX10+/PHOX2B+ ENCDCs across different developmental stages (E8 to D0). At E8, bipotent ENCDCs were common in the hindgut, co-expressing both neural crest (SOX10, PHOX2B) and lineage-specific markers (B-FABP for glia, HU for neurons). By D0, however, most cells had committed to either glial or neuronal lineages, with few remaining multipotent progenitors. These results showed a progressive restriction in their differentiation potential, consistent with previous studies in mammalian embryonic and newborn intestine where cells expressing glia and neuronal markers appear in the ENS almost immediately after colonization of the ENCDCs and, at postnatal day 0, fewer than 5% were neither neuron nor glia (Young et al., 2005; Lasrado et al., 2018; Rao and Gershon, 2018). Developmental restriction of the avian ENCDCs was further supported by our chick-*mCherry* chick recombination experiments, where E12-derived ENCDCs efficiently colonized and differentiated within the E5 ceca + hindgut, whereas D0-derived cells failed to integrate into host ganglia. These findings are consistent with earlier experiments demonstrating that the regenerative potential of ENCDCs diminishes postnatally (Hotta et al., 2010; Zhang et al., 2019), emphasizing the importance of embryonic age in stem cell-based therapies for ENS congenital disorders. When ENCDC donor quail hindgut at the wavefront level (E6 quail embryo) was recombined with preganglionic chick midgut + hindgut, intense colonization occurred, but this capacity rapidly declined with older (E10) donor hindgut (Zhang et al., 2019). In contrast, our experiments showed that when E12 chicken hindgut was recombined with preganglionic chick ceca + hindgut, the *mCherry+* chick ENCDCs migrate and extensively colonize the host hindgut, similar to those spreading during normal development. The difference in the extent of colonization between Zhang et al. (2019) and the current study may be explained by the presence or absence of the cecal region in its ability to support ENCDC migration and colonization of the hindgut.

Despite the age-dependent developmental decline in ENCDC colonization capacity, several research studies have successfully isolated and expanded ENSCs from postnatal mammalian gut tissues. These ENS-derived cells, when transplanted into transgenic animal models of Hirschsprung disease, have demonstrated the ability to survive, proliferate and migrate extensively, forming clusters of neurons and glial cells resembling enteric ganglia, and restore gut motility, indicating promising, although reduced therapeutic potential for treating congenital neurointestinal diseases (Metzger et al., 2009; Hotta et al., 2016; Cheng et al., 2017; Mueller et al., 2024). While these results show that enteric neurospheres have been well-characterized in mammalian systems and transplantation is achievable, improved methods are needed to enhance ENSC survival, proliferation, and engraftment, promote neuron and glial differentiation, and enhance neural plexus formation.

The chicken embryo is a powerful model for studying ENS development (Goldstein and Nagy, 2008). Compared to mammalian systems, the avian embryo allows easy accessibility for embryo manipulation and precise lineage tracing throughout ENS development. The developmental dynamics of ENCDC migration, proliferation, and differentiation can be specifically visualized and experimentally modulated in the chick embryo, making it an ideal experimental system for studying the cellular and molecular background of the developmental mechanisms underlying ENS formation. We therefore used the avian embryo as a complementary system to the routinely used mammalian models for testing the *in vivo* potential of enteric neurospheres in aganglionic colon. To date, three potential gut-derived cell sources have been described for the isolation of avian ENSCs and the generation of avian enteric neurospheres. Rollo et al. (2016) demonstrated the growth of SOX10+ and HU+ small aggregates (diameter range of 70–100 µm) from fluorescent-activated cell sorted E5 to E9 quail HNK1+ ENSCs cultured 1–2 days in low serum DMEM/F12 cell culture medium. Results from tissue recombination experiments (Zhang et al., 2019) show that the ability of these quail enteric aggregates to form hindgut ENS progressively declines due to the quantitative decrease of undifferentiated ENSCs, as well as to the age-related qualitative changes. ENSCs have also been isolated from E14 chicken and expanded for 2 weeks in DMEM/F12 culture medium supplemented with basic fibroblast growth factor and epidermal growth factor to form neurospheres with a diameter range of 100–150 µm (Jevans et al., 2018). The majority of the cells within these neurospheres were TUJ1 immunoreactive, and following transplantation into the spinal cord, ENSCs contribute to the host’s neural tissue. A recent study from our lab (Nagy et al., 2020) demonstrated that exogenous GDNF leads to ectopic TUJ1+ enteric aggregates (ganglioneuromas; diameter range 70–130 µm) on the surface of E6-E8 chick ceca and small intestine, and transplantation of these ganglioneuromas gives rise to a fully developed ENS in the hindgut. Interestingly, addition of GDNF to E9 chicken intestine did not result in ganglioneuroma formation, supporting the developmental decline of the GDNF effect.

In the present study, we use a growth factor mix, the GWEN supplement (GDNF, WNT11, ET-3, Noggin), that leverages the cecal molecular microenvironment that is essential for supporting hindgut ENS development. Previous studies have shown that these morphogens are highly expressed in the ceca and play synergistic roles in maintaining the undifferentiated state of ENCDCs, promoting their proliferation, and preventing premature differentiation (Druckenbrod and Epstein, 2005; Nagy and Goldstein, 2006; Nagy et al., 2021; Kurtz et al., 1994). Avian ENCDCs migrating through the ceca are mitotically strongly active in response to growth factors from the local mesenchymal environment (Nagy et al., 2021). Interestingly, while GDNF alone had minimal impact on postnatal neurospheres, consistent with prior reports in mice (Cheng et al., 2016), in our present study, the combination of GDNF with other ceca-derived growth factors (WNT11 and ET-3) promoted chicken embryonic neurosphere formation. We also show that GWEN supplementation significantly improves neurosphere yield and enhances the formation of PHOX2B+/SOX10+ enteric neural crest-rich aggregates, as well as promoting HNK1+ cell migration and TUJ1+ neurite outgrowth *in vitro*. Moreover, only GWEN-treated neurospheres formed enteric ganglia following transplantation into preganglionic chick hindgut explants, indicating that GWEN promotes not just expansion, but also functional neuronal differentiation and integration of the ENSCs. In contrast, control neurospheres contained significantly fewer HNK1 immunoreactive cells, displayed round morphology with a solid core of mesenchymal tissue, and exhibited limited contribution to the host ENS. The ability of transplanted ENSCs to populate both submucosal and myenteric plexuses and express neuronal markers (TUJ1, HU) suggests that avian neurospheres could serve as a valuable model for studying ENS repair in aganglionic conditions like Hirschsprung disease, and GWEN factors can be important to maximize the effect of cell transplantation to treat congenital neurointestinal diseases.

While our study introduces avian enteric neurospheres to ENS research, this work has limitations, and several questions remain unanswered. For example, the long-term functional integration of transplanted neurospheres, their synaptic connectivity, and their ability to restore gut motility in aganglionic models require further investigation. While chick embryos provide a powerful and accessible model for developmental studies, translational application to human ENS therapy will require adaptation of the GWEN culture method to human ENSCs, especially those derived from iPSCs. Future transcriptomic or single-cell RNA sequencing studies may clarify how this growth factor environment influences lineage differentiation trajectories and progenitor heterogeneity. In summary, our findings have significant implications for regenerative approaches to ENS diseases: i) they highlight the necessity of studying early-stage (embryonic or perinatal) ENSCs, as postnatal ENS-derived cells exhibit restricted neurogenic potential; ii) the success of GWEN media in enhancing embryonic neurosphere formation suggests that specific growth factor combinations essential for normal ENS development can optimize ENSC proliferation and differentiation for cell transplantation; iii) the avian embryo offers a cost-effective and ethically accessible system for preclinical testing of ENS regenerative cell therapies.

## Data Availability

The original contributions presented in the study are included in the article/Supplementary Material, further inquiries can be directed to the corresponding author.

## Supplementary material

SUPPLEMENTARY FIGURE S1 Characterization of developing enteric neural crest-derived cells (ENCDCs) during chicken hindgut development. Representative cross-sections of the chicken hindgut at embryonic day (E)8 **(A)**, E12 **(B)**, and day of hatching (D0; **(C)**. The boxed area in **(A)** is shown at higher magnification in panels **(A1–A5)**. Separate immunofluorescence channels show B-FABP (blue), SOX10 (white), HU (red), PHOX2B (green), and DAPI (cyan). The merged image illustrates the colocalization of SOX10+/PHOX2B + ENCDCs within developing enteric ganglia. ENCDCs differentiate into PHOX2B+/HU + enteric neurons, while B-FABP marks enteric glial precursors **(A5–C5)**.

SUPPLEMENTARY FIGURE S2 Mesenchymal derived growth factors promote neurosphere formation *in vitro*. Representative immunofluorescent images of 7-day-old neurospheres generated from E8 chicken intestine. Neuronal and glial differentiation were assessed by PHOX2B (green) and SOX10 (red) immunostaining, while proliferating cells were identified by EdU incorporation (grey). ENS-derived cells were maintained under control conditions **(A–A‴)** or cultured with various growth factor supplements to compare the individual and combinatorial effects **(C–C‴)** of GDNF, **(B–B‴)**, WNT11 **(D–D‴),** ET-3 **(E–E‴)** and Noggin **(F–F‴)**.

SUPPLEMENTARY FIGURE S3 Effects of GDNF and co-factors on proliferation, differentiation, and neurogenesis of enteric neural progenitors within neurospheres. Quantitative analysis of neurosphere formation and cellular composition under different culture conditions: control (CTRL), GDNF alone, GWEN, GDNF + WNT11, GDNF + ET-3, and GDNF + Noggin **(A)**. Number of HU+ (neuronal) cells per spheroid **(B)**. Percentage of proliferating cells (EdU+) relative to total nuclei (DAPI) **(C)**. Proportion of PHOX2B^+^ cells (% of DAPI), representing neuronal differentiation **(D)**. Percentage of PHOX2B^+^/SOX10^+^ cells (% of DAPI), indicating bipotent progenitor populations **(E)**. Percentage of proliferating PHOX2B^+^/SOX10^+^/EdU^+^ cells (% of DAPI), representing proliferating enteric neural progenitors **(F)**. Violin plots show data distributions with medians and interquartile ranges. *P < 0.05, **P < 0.01, ***P < 0.001, ****P < 0.0001, n = 8, Kruskal-Wallis test.

SUPPLEMENTARY TABLE S1 Statistical analysis of the effects of growth factor combinations on the cellular composition of neurospheres. Growth factors are listed in the column and row headers, while the analyzed cell groups are highlighted with colors. Within the table, the level of significance is shown: ns, not significant; *p < 0.05; **p < 0.01; ***p < 0.001; ****p < 0.0001. The black diagonal lines separate the analyzed cell groups. (Kruskal-Wallis test).
