# Supplementary figures and images for: Cecal growth factors promote enteric neurosphere formation and hindgut colonization in the avian model
Source: Front Cell Dev Biol. 2025 Dec 18;13:1681844. doi: 10.3389/fcell.2025.1681844 (PMC12756466; doi:10.3389/fcell.2025.1681844)

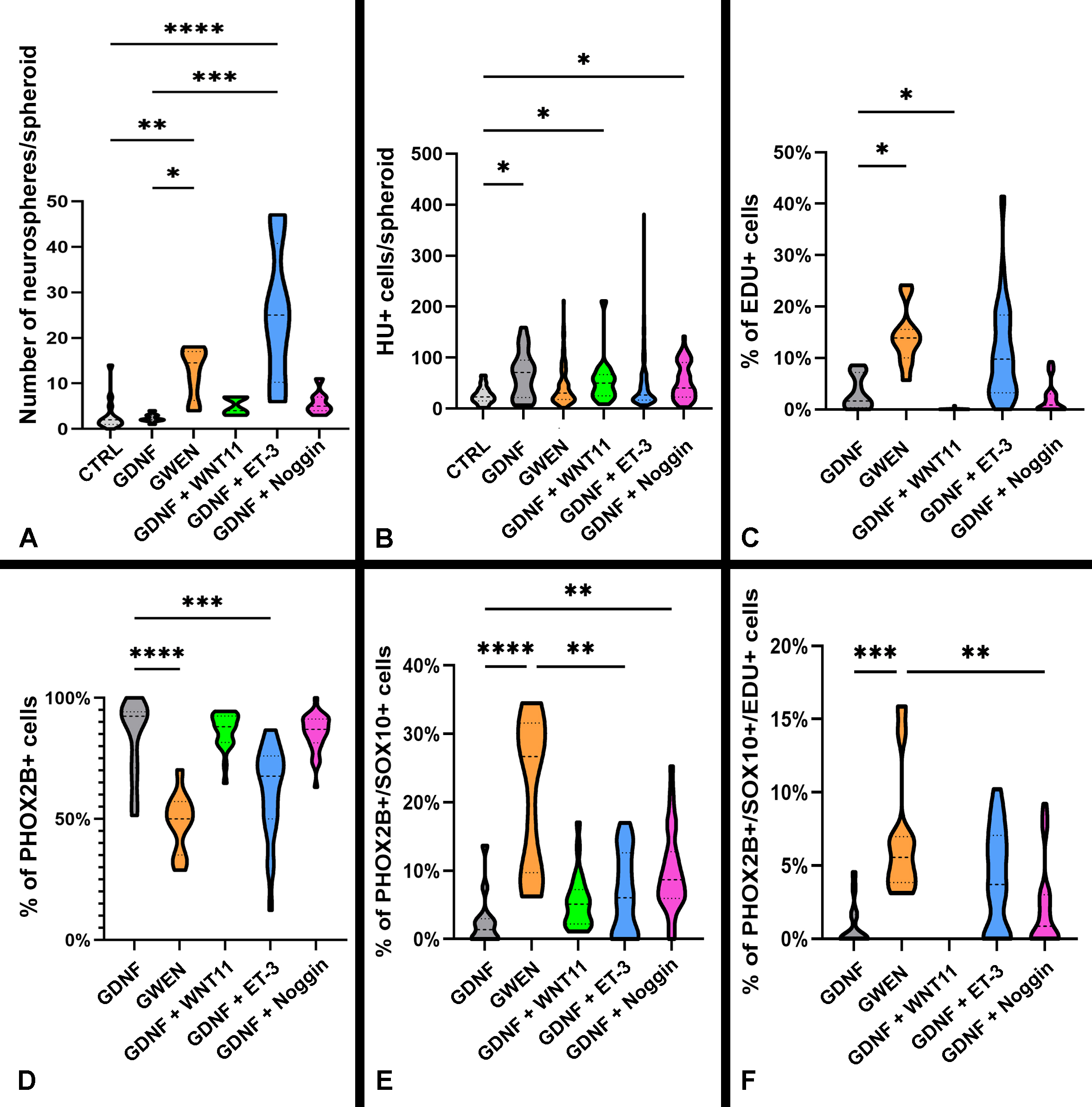

Supplement: Supplementary file 1 [file Image3.jpeg]

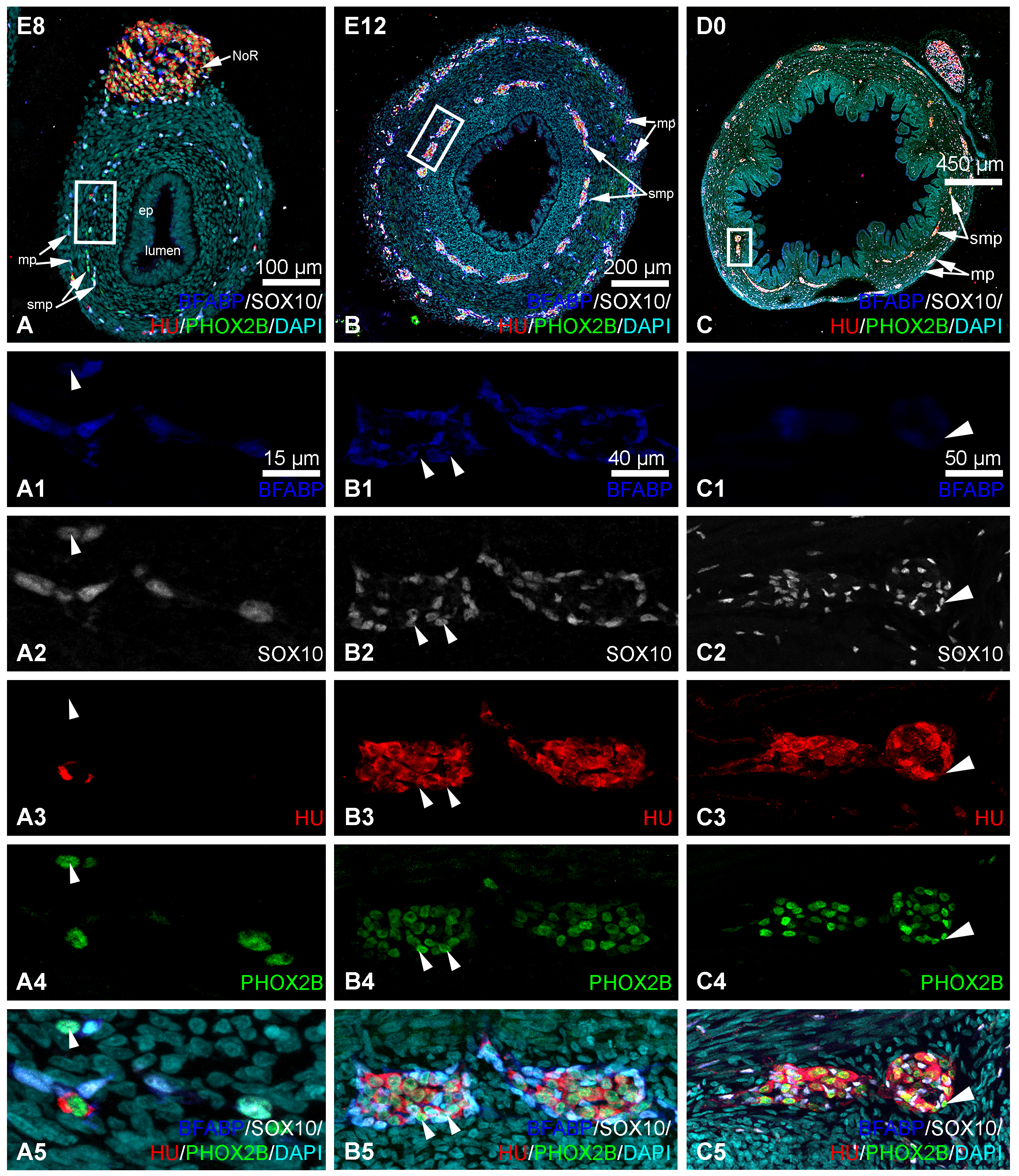

Supplement: Supplementary file 2 [file Image1.jpeg]

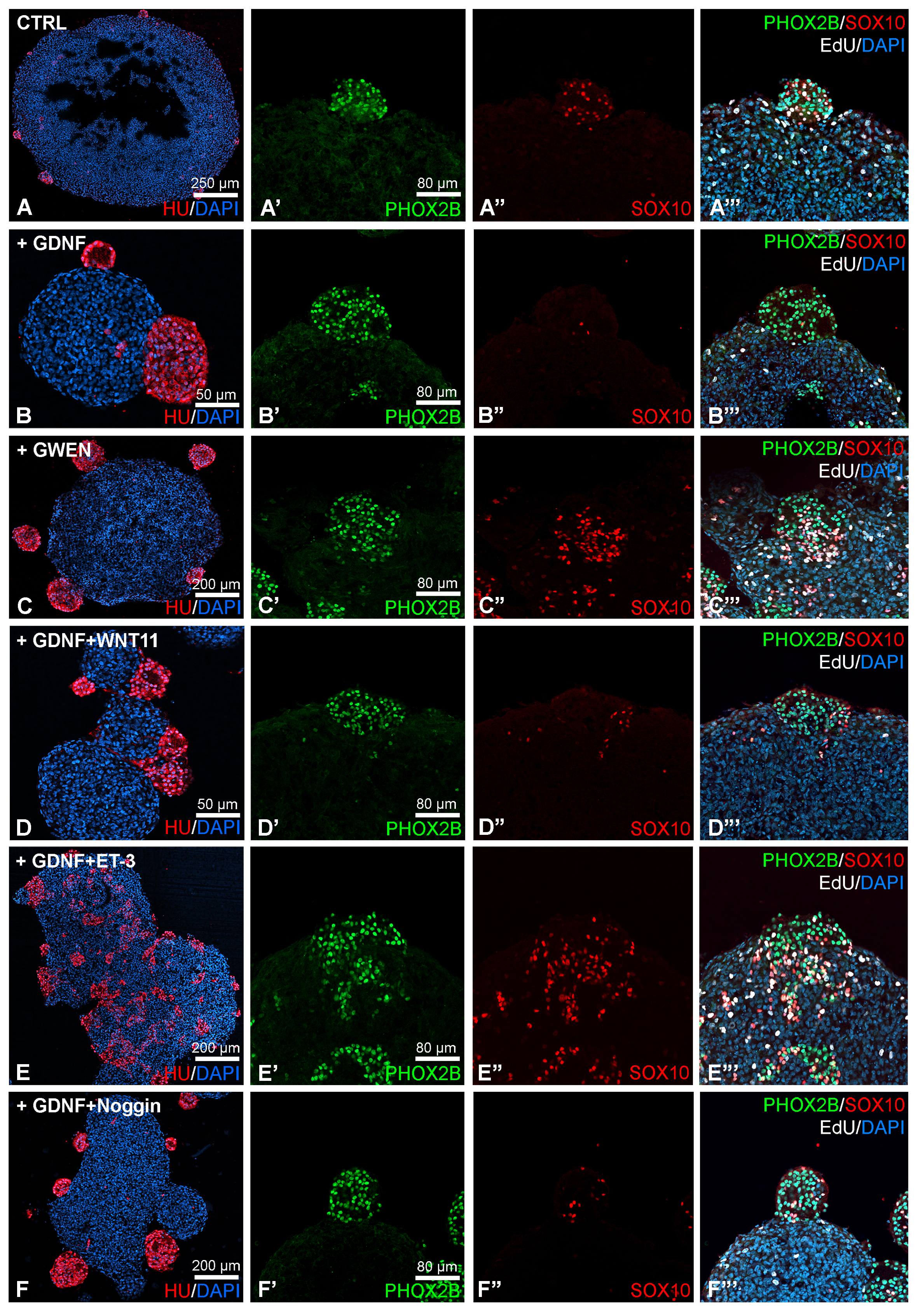

Supplement: Supplementary file 3 [file Image2.jpeg]
