# Supplementary material for: Cecal growth factors promote enteric neurosphere formation and hindgut colonization in the avian model
Source: Front Cell Dev Biol. 2025 Dec 18;13:1681844. doi: 10.3389/fcell.2025.1681844 (PMC12756466; doi:10.3389/fcell.2025.1681844)
Supplement: Supplementary file 4 [file DataSheet1.pdf]

Number of neurospheres/spheroid

	CTRL	GDNF	GWEN	GDNF+WNT11	GDNF+ET-3	GDNF+Noggin
CTRL		ns	***	ns	****	ns
GDNF	*		**	ns	****	ns
GWEN	ns	ns		ns	ns	ns
GDNF +WNT11	*	ns	ns		ns	ns
GDNF +ET-3	ns	ns	ns	ns		ns
GDNF +Noggin	*	ns	ns	ns	ns	
	CTRL	GDNF	GWEN	GDNF+WNT11	GDNF+ET-3	GDNF+Noggin

HU+ cells/spheroid

% of PHOX2B+ cells

	GDNF	GWEN	GDNF+WNT11	GDNF+ET-3	GDNF+Noggin
GDNF		****	ns	***	ns
GWEN	ns		****	ns	****
GDNF +WNT11	*	**		****	ns
GDNF +ET-3	ns	ns	ns		****
GDNF +Noggin	*	**	ns	ns	
	GDNF	GWEN	GDNF+WNT11	GDNF+ET-3	GDNF+Noggin

% of PHOX2B+/EDU+ cells

% of SOX10+ cells

	GDNF	GWEN	GDNF+WNT11	GDNF+ET-3	GDNF+Noggin
GDNF		ns	*	ns	**
GWEN	****		****	ns	****
GDNF +WNT11	ns	****		****	ns
GDNF +ET-3	ns	ns	****		****
GDNF +Noggin	ns	****	ns	**** 72.41	
	GDNF	GWEN	GDNF+WNT11	GDNF+END3	GDNF+Noggin

% of SOX10+/EDU+ cells

% of PHOX2B+/SOX10+ cells

	GDNF	GWEN	GDNF+WNT11	GDNF+ET-3	GDNF+Noggin
GDNF		****	ns	ns	**
GWEN	***		***	**	ns
GDNF +WNT11	ns	****		ns	ns
GDNF +ET-3	ns	ns	****		ns
GDNF +Noggin	ns	**	**	ns	
	GDNF	GWEN	GDNF+WNT11	GDNF+ET-3	GDNF+Noggin

% of PHOX2B+/SOX10+/EDU+ cells

% of EDU+ cells

	GDNF	GWEN	GDNF+WNT11	GDNF+ET-3	GDNF+Noggin
GDNF		*	*	ns	ns
GWEN			****	ns	****
GDNF +WNT11				****	ns
GDNF +ET-3					****
GDNF +Noggin					

* $p < 0.05$
** $p < 0.01$
*** $p < 0.001$
**** $p < 0.0001$
